# Clinical Analysis of 1,834 Colorectal Cancers

**DOI:** 10.1155/DTE.2.19

**Published:** 1995

**Authors:** Masaaki Miyaoka, Koichi Watanabe, Naoki Shimizu, Toshitaka Takeshita, Toshihiko Saito

**Affiliations:** Fourth Department of Internal Medicine Tokyo Medical College Hospital 6-7-1 Nishishinjuku Shinjuku-ku Tokyo 160 Japan

## Abstract

A total of 1,834 cases of colorectal cancers were divided into two diagnostic groups and studied. The ratio of smaller, less advanced carcinomas to the total number of colorectal cancers diagnosed by electronic videoendoscopy has increased as compared to the ratio in cases diagnosed by fiberscopy. This seems to be largely influenced by concomittent developments such as the implementation of colorectal cancer screening assisted by the popularization of immunological fecal occult blood tests and the increase in number of examinees. On the other hand, the use of the electronic videoendoscopy has been accompanied by increases in the diagnosis of minute carcinomas measuring 5 mm or less and flat carcinomas which were previously difficult to diagnose. Under these circumstances, endoscopic mucosal resection has gained popularity, widening the spectrum of therapeutic options. Nonetheless, endoscopic treatment also has been associated with increases in cases requiring laparotomy when carcinoma was found in the resected margin or those demonstrating invasion in the submucosa. This may be one result of the more aggressive application of endoscopic treatment, but the histologically recognized inability to detect carcinoma in the resected intestinal tract leaves room for improvement.


*Diagnostic and Therapeutic Endoscopy*, 1995, Vol. 2 , pp. 19–27
Reprints available directly from the publisher
Photocopying permitted by license only

© 1995 Harwood Academic Publishers GmbH
Printed in Singapore

### Clinical Analysis of 1,834 Colorectal Cancers

MASAAKI MIYAOKA, KOICHI WATANABE, NAOKI SHIMIZU, TOSHITAKA TAKESHITA and TOSHIHIKO SAITO

*Fourth Department of Internal Medicine, Tokyo Medical College Hospital, 6-7-1 Nishishinjuku Shinjuku-ku, Tokyo 160, Japan*

*(Received December 27, 1993; in final form March 14, 1995)*

A total of 1,834 cases of colorectal cancers were divided into two diagnostic groups and studied. The ratio of smaller, less advanced carcinomas to the total number of colorectal cancers diagnosed by electronic videoendoscopy has increased as compared to the ratio in cases diagnosed by fiberscopy. This seems to be largely influenced by concomittent developments such as the implementation of colorectal cancer screening assisted by the popularization of immunological fecal occult blood tests and the increase in number of examinees. On the other hand, the use of the electronic videoendoscopy has been accompanied by increases in the diagnosis of minute carcinomas measuring 5 mm or less and flat carcinomas which were previously difficult to diagnose. Under these circumstances, endoscopic mucosal resection has gained popularity, widening the spectrum of therapeutic options. Nonetheless, endoscopic treatment also has been associated with increases in cases requiring laparotomy when carcinoma was found in the resected margin or those demonstrating invasion in the submucosa. This may be one result of the more aggressive application of endoscopic treatment, but the histologically recognized inability to detect carcinoma in the resected intestinal tract leaves room for improvement.

**KEY WORDS:** electronic videoendoscopy, colorectal cancers, endoscopic resection, endoscopic mucosal resection

### INTRODUCTION

Recent years have seen an increase in colorectal cancers in Japan. Changes in eating habits, especially those centering on high-fat low-fiber diets have be incriminated as causative factors. As a countermeasure to lower the associated fatal cases, colorectal cancer screening based on the immunological fecal occult blood test has been widely implemented. This, coupled with advances in diagnostics centering on electronic videoendoscopy, has achieved a remarkable improvement in the rate of early detection of colorectal cancers in recent years. This paper deals with a comparison of colorectal cancers diagnosed by the currently increasingly popular fourth generation endoscope, the electronic videoendoscope, as compared with colorectal cancers diagnosed by fiberscopes.

Address for Correspondence: Msaaki Miyaoka, M.D., Fourth Department of Internal Medicine, Tokyo Medical College Hospital, 6-7-1 Nishishinjuku Shinjuku-ku, Tokyo 160, Japan.

### MATERIALS AND METHODS

The subjects comprised a total of 1,834 cases encountered at the Fourth Department of Internal Medicine, Tokyo Medical College Hospital, during the 24-year period from January 1969 to August 1993. They consisted of 1,232 males and 602 females. Their ages ranged from age 16 to 91, averaging 60.2 years of age. Of these cases, 1779 cases underwent colorectoscopy. From February 1987, fiberscopy began to be replaced by electronic videoendoscopy. The former method diagnosed 653 colorectal cancers as opposed to the 1,126 cases diagnosed to date by the latter. We collected all evaluable colorectal cancer cases and compared the two types of instruments in terms of the following: The items of evaluation are 1) change in the number of colorectal cancer cases, 2) age, 3) sex, 4) site, 5) causes for detection of colorectal cancers, 6) size, 7) degree of penetration, 8) shape, 9) ratio of sm cancer viewed by size and shape, 10) endoscopic therapy and 11) cure rate of endoscopic therapy. Both the diagnostic criteria for, and classification of colorectal cancer complied with the General Rules for Clinical and Pathological Studies on Cancers of the Colon, Rectum and Anus.[1]

### RESULTS

### 1) Change in the number of colorectal cancer (Fig. 1)

The incidence of colorectal cancers began to rise in number from 1981 to 1983, and sharply increased in 1987 and 1989. Early cancers began to be seen from 1972–1974, their proportions rose from 1984 to 1986, constituting nearly 50% of all cases from 1990 to 1992 and finally exceeding 60% of all colorectal cancer cases in 1993.

### 2) Age (Fig. 2)

Evaluation of the age distribution in the 1,834 cases of colorectal cancers revealed a predominance of those aged from 50 to 69 years (59.5%). When the cancers are broken down into early and advanced cancers, early cancers were most frequently seen in subjects in their 50's, while advanced cases were most frequently seen in individuals in their 60's.

The number of cases diagnosed by electronic videoendoscopy rather than fiberscopy increased in individuals aged 40–59 years and also in those aged 80 years or older. The proportion of advanced cancers diagnosed by electronic endoscopy was higher in subjects in their 50's and those aged 80 years or older, while that of the early cancers similiarly diagnosed was higher in individuals in their 40's.

### 3) Sex (Fig. 3)

Males outnumbered females by 1.16:1 overall and 3.17:1 for early cancers. Evaluation by instrument type reveals that more male cases of colorectal cancers were diagnosed by electronic endoscopy rather than fiberscopy, and the trend was even more pronounced in early cancers.

### 4) Site (Fig. 4)

Rectal cancers and sigmoid colon cancers accounted for 65% of all cancers. When a distinction was made between advanced cancer and early cancer, the proportions of sigmoid colon cancer and descending colon cancer were higher in early cancers.

Evaluating detection according to type, a slight decline was noted in the ratio of rectal cancers and sigmoid colon cancers diagnosed by electronic videoendoscopy compared to those diagnosed by fiberscopy. Similarly, a small increase was noted in the number of electronic videoendoscopy diagnosed cancers of the transverse and ascending colon, although no significant differences were seen between fiberscopy and electronic videoendoscopy. The findings remained virtually unchanged concerning evaluations done in terms of advanced cancer vs. early cancer.

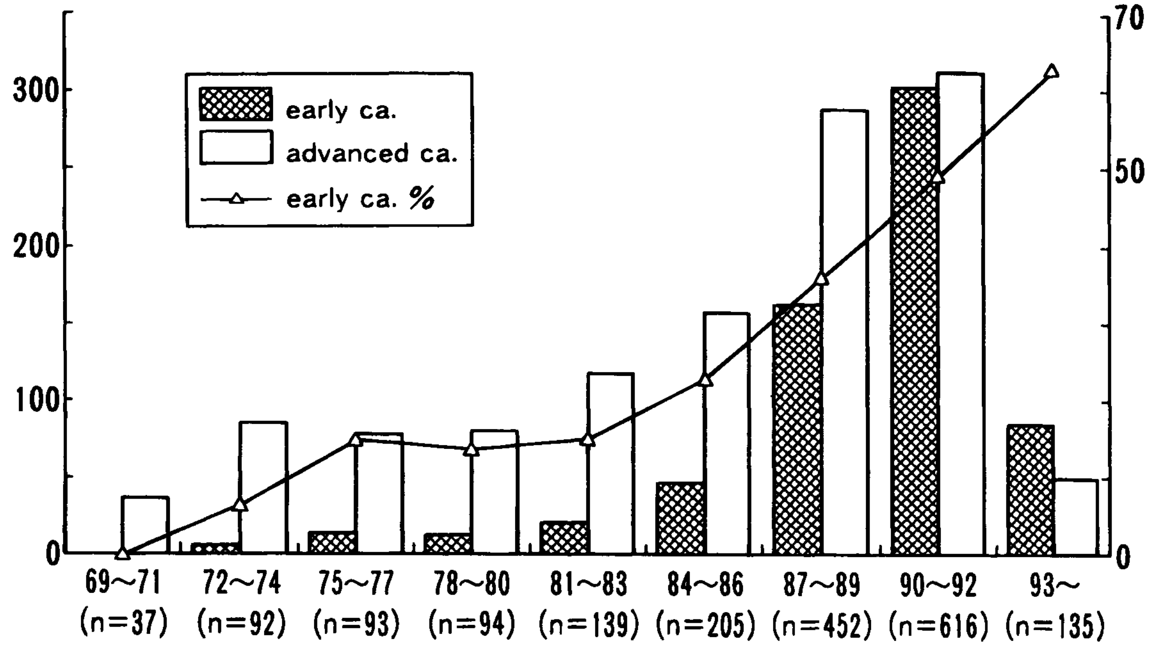

**Figure 1** Changes in the Number of Colorectal Cancer Cases.

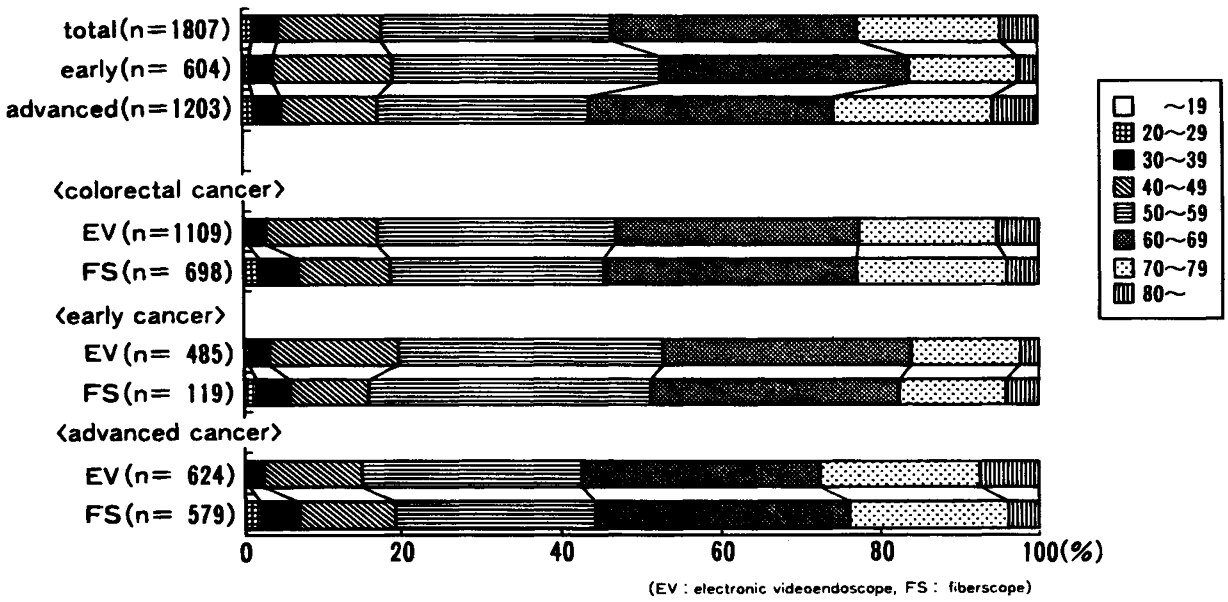

**Figure 2** Age

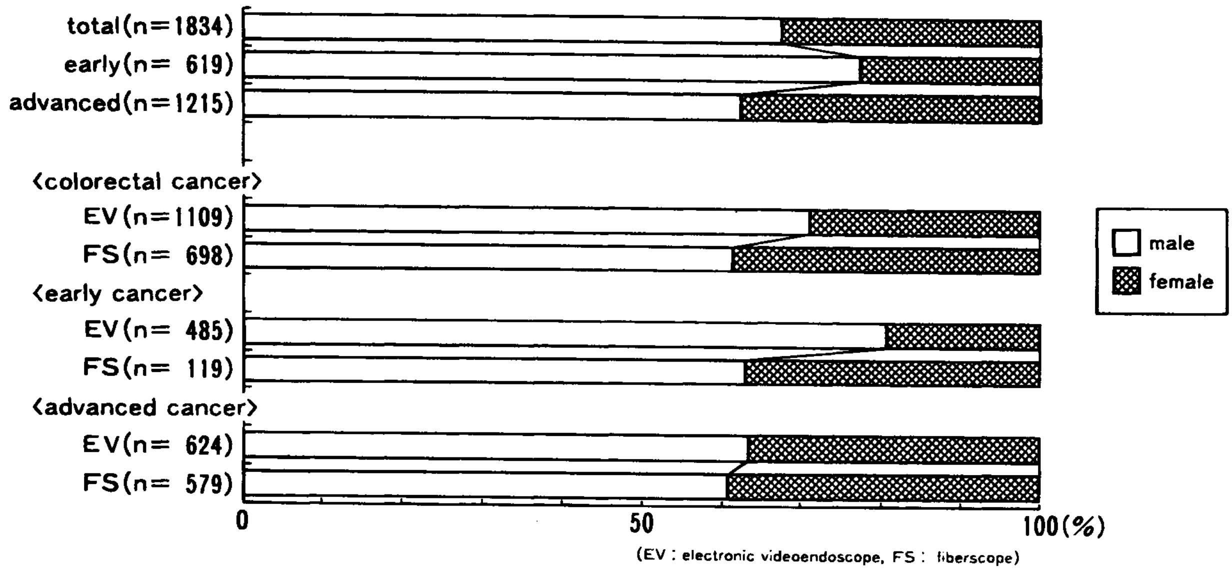

**Figure 3** Sex

As far as early cancer was concerned, there was a striking increase in sigmoid cancer.

**5) Causes for detection of colorectal cancers (Fig. 5)**

In all cases of colorectal cancer, the predominant symptoms were macroscopic bleeding and abdominal pain. However, when divided into advanced cancer and early cancer, the proportions detected by occult bleeding or during follow-up were higher in early cancer cases, whereas such cases detected as a result of macroscopic bleeding and abdominal pain decreased markedly.

With diagnosis by electronic vidoendoscopy, the proportion of macroscopic bleeding and abdominal pain as causes for the detection of colorectal cancers decreased and there were prominent increases in the proportion of cases detected by occult bleeding or during follow-up compared to fiberscopy. These trends were most remarkable when it comes to early cancers.

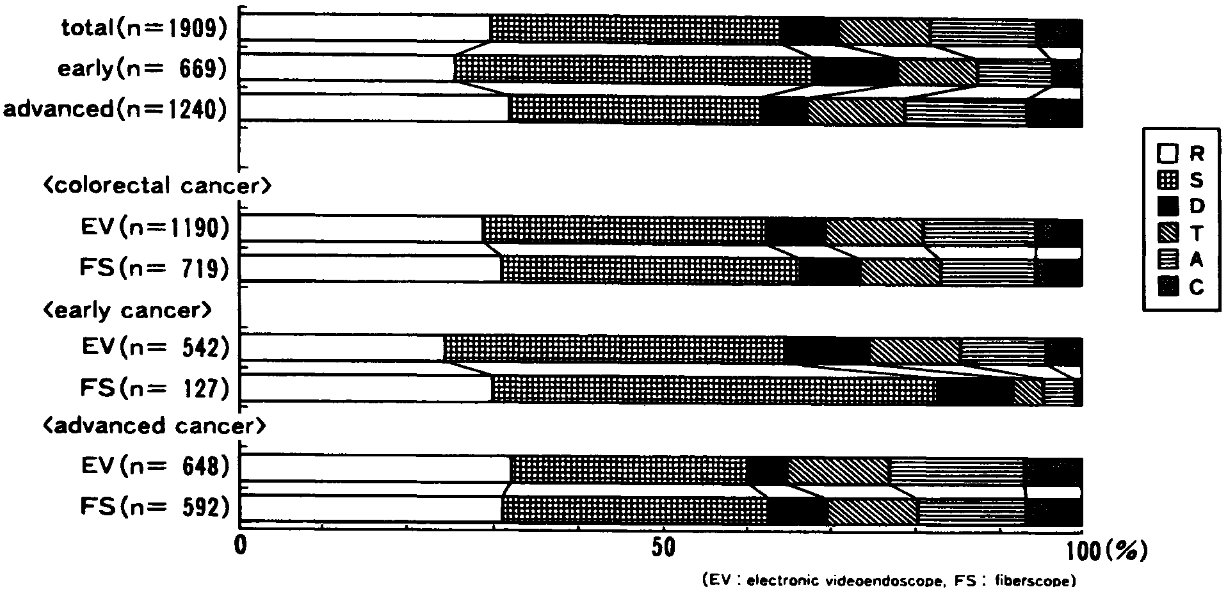

**Figure 4** Site

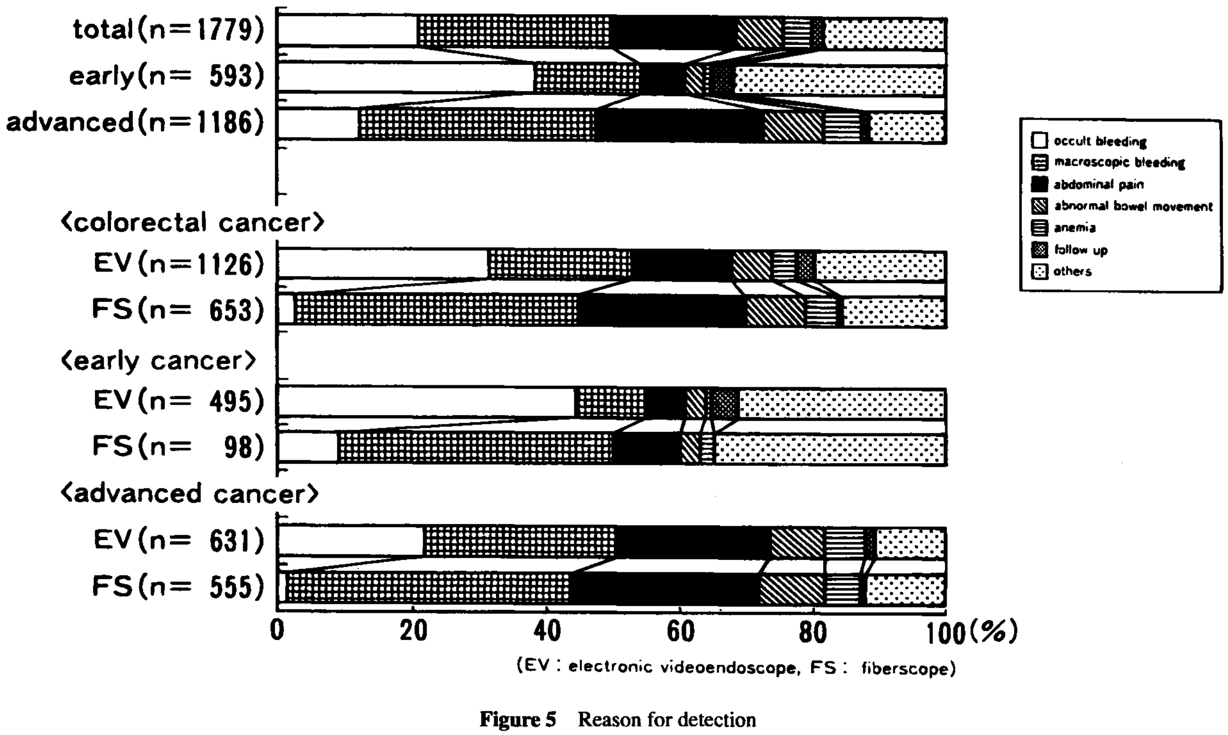

**Figure 5** Reason for detection

### 6) Size (Fig. 6)

Most colorectal cancers were 11–20 mm, the next most common size being 10 mm or less. This tendency is especially pronounced with early cancers. The smallest size of advanced cancer was 11 mm, the most common size being 41–60 mm, constituting 44% of all advanced cancers.

Electronic videoendoscopy detected more relatively small colorectal cancers measuring less than 20 mm than fiberscopy. In particular, the diagnosis of minute cancers measuring 5 mm or less is markedly better by electronic video-endoscopy than fiberscopy.

### 7) Degree of penetration (Fig. 7)

As for evaluation by degree of invasion, m cancers head the list, followed in descending order by ss cancers, and and sm cancers.

It is of note that among cancers diagnosed by electronic videoendoscopy the ratio of cancers confined to the mucosa propria and pm lesion increased in proportion to the total number of colorectal cancers as compared to the ratio seen in fiberscopically diagnosed lesion. However, the ratio of sm lesions or lesions invading the serosa decreased.

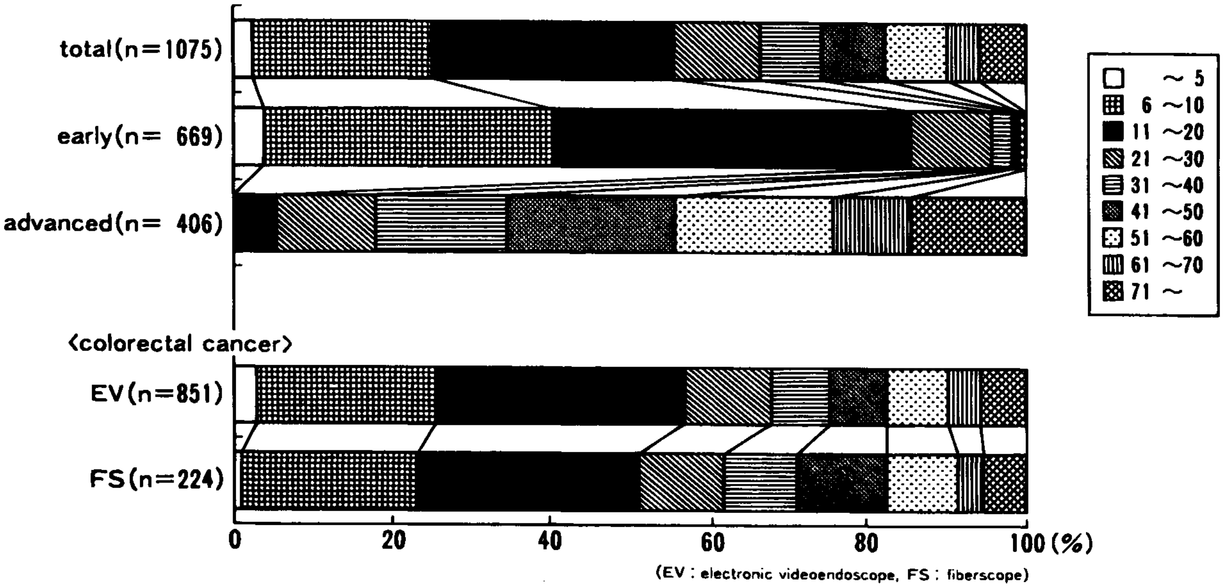

**Figure 6** Size

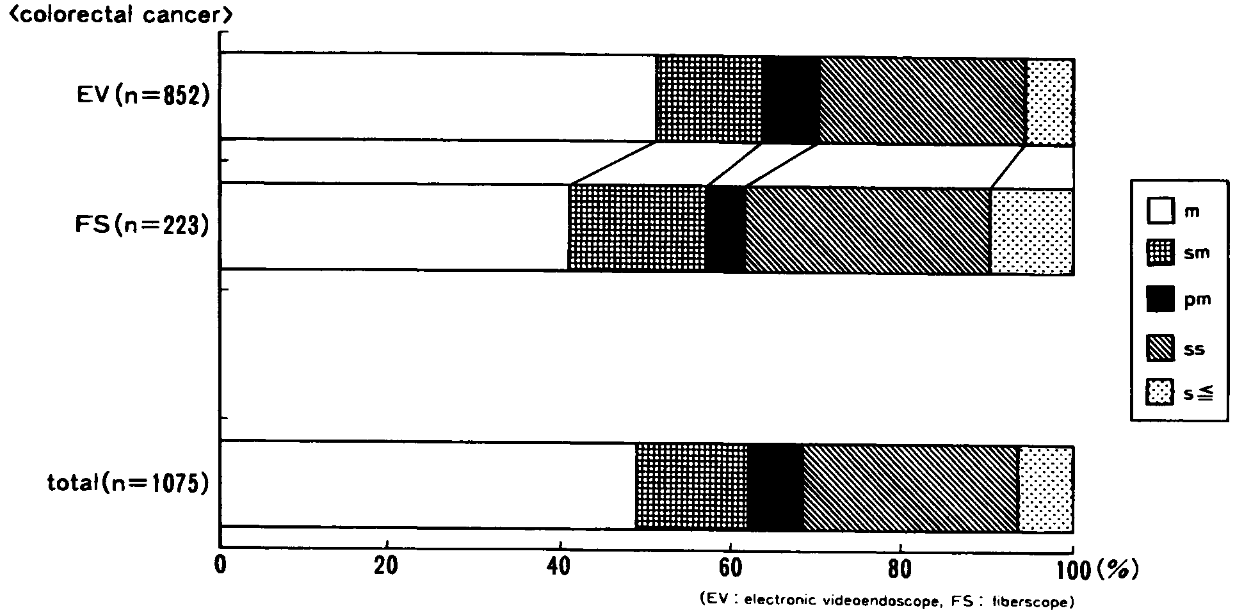

**Figure 7** Degree of penetration

### 8) Shape (Fig. 8)

Type 2 (ulcerative type) was the most common overall followed by type Ip. When distinguishing between early cancer and advanced cancer, the Ip type and Isp type make up the vast majority, in the early cancer category, while type 2 was by far the most common type of the advanced cancer.

In colorectal cancers diagnosed by electronic videoendoscopy, the proportions of type Ip, type Isp, type IIa, type IIc early cancer and type 1 (polypoid type) advanced cancer increased, while those of IIa+IIc early cancer and type 2 advanced cancer declined.

### 9) Proportion of sm cancer viewd by size and shape (Table 1)

Regardless of shape, the larger the size, the greater the proportion of sm cancer. Sm cancer was even been demonstrated in a type Isp foci 5 mm or less in size. Higher proportions of sm cancer were seen in flat type lesions with a central depression such as type IIa+IIc, IIc and IIc+IIa, but the proportion of sm lesions was lower in type IIa-v, even when of a larger size.

### 10) Endoscopic theraphy (Fig. 9)

With both types of instruments, snare polypectomy was the most common procedure, being performed in 77% of all cases. In electronic videoendoscopy, the necessity for conventional surgery resulting from hot biopsy or biopsy procedures has decreased, while endoscopic mucosal resection and piecemeal polypectomy increased.

### 11) Endoscopic theraphy cure rates

Electronic videoendoscopy has a higher cure rate than fiberscopy in the flat type, but both types have poor results in other type lesions (Table 2).

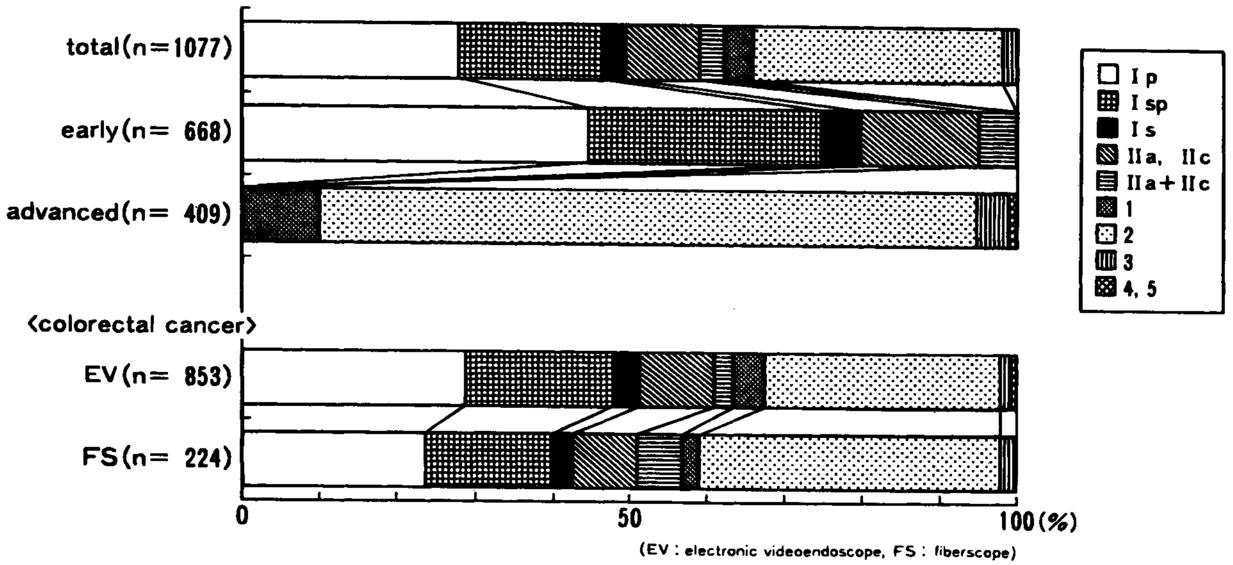

Figure 8 Shape

Table 1 Proportion of sm Cancer According to Size and Shape

| Size | *Ip* No. | *Ip* sm (%) | *Isp* No. | *Isp* sm (%) | *Is* No. | *Is* sm (%) | *IIa* No. | *IIa* sm (%) | *IIa–v* No. | *IIa–v* sm (%) | *IIa + IIc IIc, IIc + IIa* No. | *IIa + IIc IIc, IIc + IIa* sm (%) | *Total* No. | *Total* sm (%) |
|---|---|---|---|---|---|---|---|---|---|---|---|---|---|---|
| – 5 | 0/5 | 0 | 1/10 | 10.0 | 0/12 | 0 | | | | | | | 1/27 | 3.7 |
| 6–10 | 9/84 | 10.7 | 18/101 | 17.8 | 1/14 | 7.1 | 5/33 | 15.2 | 0/2 | 0 | 8/13 | 61.5 | 41/247 | 16.6 |
| 11–20 | 27/181 | 14.9 | 24/67 | 35.8 | 3/10 | 30.0 | 4/30 | 13.3 | 0/8 | 0 | 9/15 | 60.0 | 67/311 | 21.5 |
| 21–30 | 8/27 | 29.6 | 8/19 | 42.1 | 0/1 | 0 | 4/6 | 66.7 | 0/9 | 0 | 7/7 | 100 | 27/69 | 39.1 |
| 31– | 1/6 | 16.7 | 0/5 | 0 | | | 0/2 | 0 | 4/13 | 30.8 | 1/1 | 100 | 6/27 | 22.2 |
| Total | 45/303 | 14.9 | 51/202 | 25.2 | 4/37 | 10.8 | 13/71 | 18.3 | 4/32 | 12.5 | 25/36 | 69.4 | 142/681 | 20.9 |

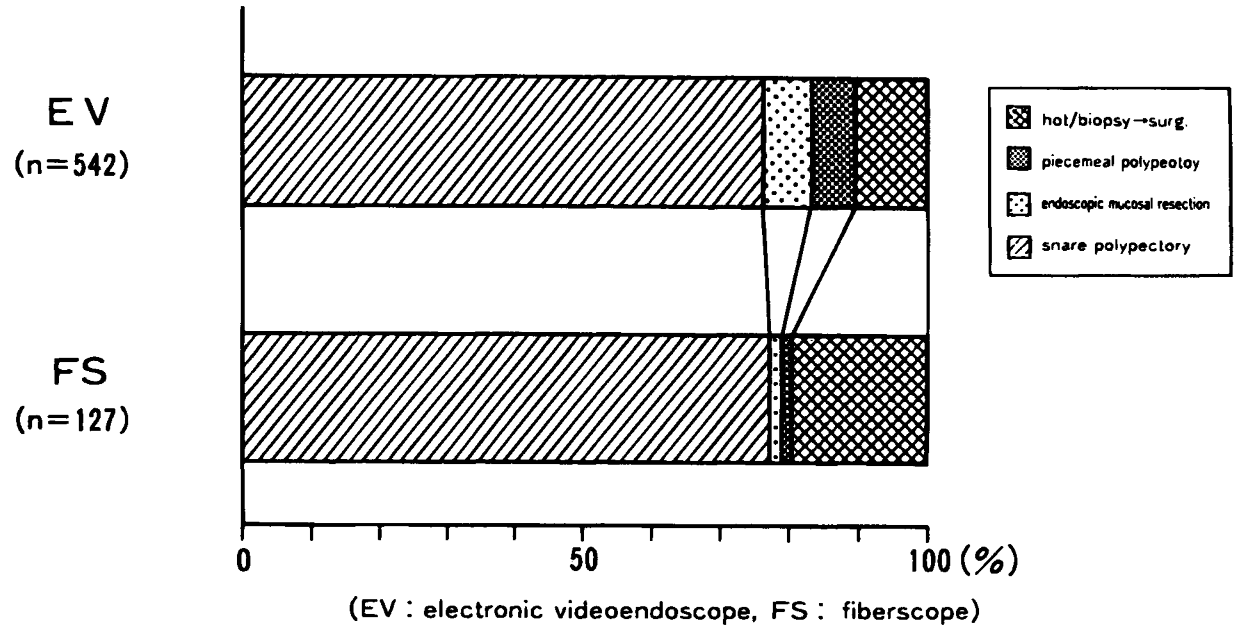

**Figure 9** Endoscopic therapy

**Table 2** Endoscopic Therapy Cure Rates

*Electronic videoendoscope*

| | *Ip* | | *Isp* | | *Is* | | *IIa* | | *IIa-v* | | *IIa + IIc IIc,IIc + IIa* | | *Total* | |
|---|---|---|---|---|---|---|---|---|---|---|---|---|---|---|
| *Size* | *No.* | *%* | *No.* | *%* | *No.* | *%* | *No.* | *%* | *No.* | *%* | *No.* | *%* | *No.* | *%* |
| - 5 | 3/3 | 100 | 5/6 | 83.3 | 2/2 | 100 | | | | | | | 10/11 | 90.9 |
| 6–10 | 59/69 | 85.5 | 58/73 | 79.5 | 7/10 | 70.0 | 15/22 | 68.2 | | | 2/9 | 22.2 | 141/183 | 77.0 |
| 11–20 | 129/146 | 88.3 | 31/56 | 55.4 | 6/8 | 75.0 | 13/19 | 68.4 | 5/5 | 100 | 2/3 | 66.7 | 186/237 | 78.5 |
| 21–30 | 18/20 | 90.0 | 10/17 | 58.8 | | | 1/3 | 33.3 | 2/2 | 100 | 0/2 | 0 | 31/44 | 70.5 |
| 31– | 3/4 | 75.0 | 1/3 | 33.3 | | | | | 1/1 | 100 | | | 5/8 | 62.5 |
| Total | 212/242 | 87.6 | 105/155 | 67.7 | 15/20 | 75.0 | 29/44 | 65.9 | 8/8 | 100 | 4/14 | 28.6 | 373/483 | 77.2 |

*Fiberscope*

| | *Ip* | | *Isp* | | *Is* | | *IIa* | | *IIa–v* | | *IIa + IIc IIc, IIc + IIa* | | *Total* | |
|---|---|---|---|---|---|---|---|---|---|---|---|---|---|---|
| *Size* | *No.* | *%* | *No.* | *%* | *No.* | *%* | *No.* | *%* | *No.* | *%* | *No.* | *%* | *No.* | *%* |
| 6–10 | 13/14 | 92.9 | 22/23 | 95.7 | 2/2 | 100 | 4/6 | 66.7 | 1/1 | 100 | 0/2 | 0 | 42/48 | 87.5 |
| 11–20 | 26/28 | 92.8 | 8/11 | 72.7 | | | 0/2 | 0 | 0/1 | 0 | 0/1 | 0 | 34/43 | 79.1 |
| 21–30 | 6/7 | 85.7 | 1/1 | 100 | | | | | | | | | 7/8 | 87.5 |
| 31– | 1/2 | 50.0 | 1/1 | 100 | | | | | | | | | 2/3 | 66.7 |
| Total | 46/51 | 90.2 | 32/36 | 88.9 | 2/2 | 100 | 4/8 | 50.0 | 1/2 | 50.0 | 0/3 | 0 | 85/102 | 83.3 |

In our investigation of 51 patients who had subsequently undergone laparotomy after initial endoscopic treatment, we found cancers remaining in the resected intestinal tract in 10 cases (19.6%), and in 15 cases (29.4%), the intestinal resection was necessitated becase of residual cancer on the polypectomy margin.

### DISCUSSION

The conventional fecal occult blood test was a chemical procedure with questionable sensitivity and specificity, and low reliability. With an eye to applying the immunological fecal occult blood test to screening of colorectal cancer, we developed a fecal occult blood test with human hemoglobin antibody and confirmed its usefulness, thereby bringing the latex method (OC Hemodia®) to perfection.[2] Detection of colorectal cancer cases steadily increased since 1984 as these techniques gained increasing popularity. The number of colorectal cancers detected by positive immunological fecal occult blood test has grown since 1987 when electronic videoendoscopy was introduced. Since most large, deeply penetrating colorectal cancers are positive on the immunological fecal occult blood test,[3] many of these colorectal cancers have been diagnosed by electronic videoendoscopy. The immunological fecal occult blood test is also capable of yielding a high positive rate in pedunculated foci. This seems to be responsible for the increase in detection of colorectal cancers with pedunculated foci. Higher proportions of colorectal cancers were diagnosed by electronic videoendoscopy in patients aged 40–59 years and in men rather than women. These data are probably partly due to a pick-up bias arising from workplace screening, in addition to the specificity of the disease. Therefore, to interpret these data as meaning an increase in colorectal cancers among young men would be unjustified. Although recent years have seen a dramatic increase in the numbers of working women in Japan, the number of working women in age groups with high cancer incidence is low. Thus, the observed increase in colorectal cancers are greatly influenced by social background, especially in early cancers. The fact that those aged 80 years or older have higher incidence of colorectal cancer is partly related to increased longevity.

The most common location of colorectal cancer is the sigmoid colon, perhaps as a result of increased detection of type Ip early cancers in the sigmoid colon that became more easily detected with the developement of flexible colonoscopes.

Undoubtedly the immunological fecal occult blood test has made a significant, contribution, but it is of limited ability in the detection of flat lesions, many of which are sm cancers, even if small. The test yields a positive rate of approximately 40% for early cancers,[3] but it is certainly insufficient as a means of screening for early cancer. The main target of colorectal cancer screening is Dukes' B type lesions, which increased detection of cancer measuring 40 mm or less and pm depth of invasion is an encouraging sign, we must also recognize that the possibility of this test that can be successfully treated by endoscopic therapy is low.

More recently, early cancers have been increasingly detected in follow-up after endoscopic therapy for cancers and adenomas, emphasizing the importance of follow-up and careful post-therapeutic observation. Incidentally, a total of 143 cases of multiple colorectal cancers have been detected at our department.

With the fiberscope we had to peer through the small eyepiece lens, but with the electronic videoendoscope we obtain a bright, large picture. The latter offers several advantages such as easier detection of minute lesions, easier staff coordination, less fatigue and educational merits. When it comes to the diagnosis of colorectal cancers, the influence of electronic videoendoscopy has not necessarily been great. Nevertheless, the increases in cases of flat lesions such as IIa and IIc type early cancers and minute cancers diagnosed by electric videoendoscopy deserve attention. This also applies to adenomas. Electronic videoendoscopy seems to have definitely upgraded the diagnostic capability with regard to minute lesions. In addition, washing of the intestinal tract has contributed remarkably to the improved preprocedural preparation of right-side areas such as the ascending colon and cecum.[4] Indeed, detection of minute lesions at the site has become much easier as a result.

The increased detection of flat lesions by electronic videoendoscopy has brought about a new technique in endoscopic therapy. Specifically, flat lesions are devoid of a boundary with the normal mucosa so that the snare wire may easily slip. This causes difficulty in strangulating the whole lesion, which increases the risk of leaving part of the focus intact. In this case, an endoscopic mucosal resection is performed after submucosal injection of hypertonic glucose solution or physiological saline, to cause elevation of the lesion to facilitate the placing of the snare wire on the lesion. This procedure enables safe resection of the lesion, but is limited. When applied to large lesions, it may leave a residual positive margin and result in more laparotomies among the cases in which endoscopic treatment had been attempted. Therefore, modified resection should be applied to small foci. One definite advantage of this technique is that when no elevation of the lesion is recognized following the injection of the solution, it indicates the presence of massive invasion of the submucosal layer, and therefore the need for conventioned open surgery. Elsewhere, an increase in minute cancers has raised problems concerning hot biopsy therapy. Although simple, hot biopsy is a debatable procedure because the condition of the cut end is unclear.

The advent of electronic videoendoscopy has been accompanied by an increase in the number of endoscopically treated cases requiring conventional surgery. This seems to be due to the widened indications of endoscopic resections. In our investigation of 51 patients who had subsequently undergone laparotomy after initial endoscopic treatement, we found cancers remaining in the resected intestinal tract in 10 cases, or 19.6% of all cases. In 15 cases, or 29.4% of these cases, the intestinal resection was necessitated because of residual cancer on the polypectomy margin. Therefore improvement in the endoscopic procedure application of the procedure should requires further refinement of the procedure.

Since the electronic videoendoscope constructs an image through electric signals, the image can be processed by computers. We have performed studies on enhanced imaging and quantification for some time. In image processing, a dye-spray method can be used to improve the detection of minute lesions, but, it would be difficult to spray-dye the whole intestinal tract. Dye-spraying should be done after detection of a lesion or abnormality, and would be difficult to performed throughout the entire colon as a screening method to improve detection. We attempted a method of image processing designed to make the endoscopic picture more intelligible through enhancement of minute irregularities and color tone. Structural enhancement by band pass filter intensity in HSI (hue, saturation, intensity) color space and color enhancement by histogram flattening of saturation (S) in HSV (hue, saturation, value) color space was attempted. Based on the results from a number of images,[5] we made a band pass filter providing good images on an experimental basis and used it in the clinical setting. As a result, detection of flattened and minute lesions was improved over ordinary electronic videoendoscopy, but regrettably there was no statistically significant improvement with regard to the detection of cancers. As such, extensive observation of the surface structure seems necessary to decide whether or not resection should be carried out. The results of color enhancement were rather discouraging as it varied, depending on the distance from the object observed, and reproducibility could not be guaranteed.

In addition, we are attempting quantification employing images taken by a dissecting microscope for a more objective assessment of an endoscopic picture. This study is based on the fact that in cases of tumors, the pit pattern of the surface becomes more complicated compared to areas of dysplasia, and we are addressing the question of image quantification by referring to the maximal diameters and degrees of circularity of the pits. At this stage, the ordinary fiberscope fails to allow observation of the surface structure in all types of polyps. Its resolution in cases of small foci is not sufficiently high, thereby necessitating extensive observation by the electronic videoendoscope with the aid of the high density picture element charge coupled device (CCD).

The resolution of the electronic endoscope will improve as the number of CCD picture elements increases. Four hundred thousand elements are the limit for television monitors currently available, and more elements are possible only using a Hi-Vision monitor.

Since a flattened lesion, if not large, is more frequently a deep-penetrating focus, close observation is essential. Although more accurate observation is possible with the advent of the electronic videoendoscope, there remains unsolved blind spots inherent in the endoscope such as areas behind the semi-lunar folds and inside the flexures. As such, many features of the endoscope must be modified. This includes not only the CCD, but also the angulation characteristics and introduction of a wide-angle observation lens.

### REFERENCES

1. General rules for clinical and pathological studies on cancer of colon, rectum, anus. The 3rd. edition. Japanese Research Society for Cancer of Colon and Rectum, Kanehara Syuppan KK, Tokyo, 1985.
2. Takeshita T, Horiguchi J, Miyaoka M, et. al. Latex agglutionation test for fecal occult blood. J Jpn Soc Colo-proct 1985;38:780–783.
3. Chen PC. Clinical study on latex agglutionation reaction for immunological fecal occult blood test. J Tokyo Med Coll 1988;46:269–278.
4. Davis GR, Santa Ana CA, Morawski SG et al. Development of a lavage solution associated with minimal water and electrolyte absorption or secretion. Gastroenterology 1980;78:991–995.
5. Horiguchi J. A clinical study of image processing in electronic endoscopy of lower intestinal tract-structure and color enhancement. J Tokyo Med Coll 1990;48:607–618.

